# The Effect of an Educational Plan Based on the Roy Adaptation Model for Fatigue and Activities of Daily Living of Patients with Heart Failure Disease

**DOI:** 10.4314/ejhs.v30i4.11

**Published:** 2020-07-01

**Authors:** Mahdieh Abdolahi, Mohammad Mahdi Doustmohamadi, Hojjat Sheikhbardsiri

**Affiliations:** 1 Department of Nursing, Zarand branch, Islamic Azad University, Zarand, Iran; 2 Social Determinants of Health Research Center, Institute for Futures Studies in Health, Kerman University of Medical Sciences, Kerman, Iran

**Keywords:** Roy model, heart failure, fatigue, daily activities

## Abstract

**Background:**

Cardiac failure is one of the most common chronic diseases with high rate of morbidity and mortality. Fatigue and decreased ability to perform daily activities are of the most common complications of this disease. The purpose of this study was to determine the effect of an educational plan based on Roy adaptation model on fatigue and daily activities in patients with heart failure.

**Methods:**

This experimental study was performed on 60 heart failure patients admitted in two educational hospitals supervised by the Kerman University of Medical Sciences in 2019. Sample was randomly assigned into two intervention and control groups. The intervention group received the care plan through a face-to-face and group training program in 6 sessions at one month. Interval followed by a follow-up period for 4 weeks later. Fatigue level was calculated based on piper fatigue scale, and daily activities were calculated by Barthes scale.

**Results:**

The finding indicated that there was a significant difference between the two groups (control and intervention) after the intervention. The result showed that the intervention group had significantly lower mean scores in fatigue and higher mean scores in daily activities compared to the control group after intervention P ≤ .05. There was a significant relationship between fatigue and daily activities of life with frequency of hospitalization and duration of disease in both groups, P ≤ .05.

**Conclusion:**

Implementation of Roy model-based education program as a low-cost, effective, and non-aggressive nursing intervention can reduce fatigue, and improve daily activities in patients with heart failure.

## Introduction

Heart diseases are one of the most prevalent chronic diseases and one of the main causes of mortality worldwide. Meanwhile, heart failure is regarded as the common ultimate path for all cardiac disorders (1). Prevalence of heart failure is increasing in the world (2). According to reports for American Heart Association in 2016, it is estimated that currently 5.7 million individuals in USA live with heart failure and annually 5500000 ones are caught by this disease (3). Heart failure is also regarded as a major mortality factor in Iran, and by changing the age pyramid of society and population aging, in the near future, the current outbreak will be 3,500 patients per one hundred thousand people (4). It is estimated that between 30-56.6 percent of patients with heart failure are re-admitted to the hospital at the first trimester after admission (5).

Heart failure, in addition to imposing heavy costs on the individual and the community, has many complications and side effects for the patient. Often, these patients experience symptoms such as dyspnea, fatigue, sleep disturbances, depression, and chest pain, which limit the patient's daily social and physical activity and reduce the quality of life (6). In the meantime, fatigue is one of the most common and debilitating symptoms in heart failure (5), which is often neglected because the relatives and the treatment team treat it as an ambiguous and indeterminate complaint. Physically, fatigue is absence of energy , cognitively it is a defect in senses concentration, and emotionally it is a decrease in motivation or interest(3). Prevalence of fatigue in heart failure varies between 50 – 96 percent, and it is associated with increased mortality risk in patients with heart failure by 2–3 times (1).

Fatigue and non-tolerance of activity in these patients causes loss of independence in performing common life activities, and makes them dependent on others in self-care. Thus, it influences quality of their life. Therefore, paying attention to fatigue in patients is highly important in heart failure management programs (7). Meanwhile, nurses often are in the best situation for identifying symptoms of fatigue and investigating the effect of these symptoms on daily performance, interpersonal relationships, and quality of life of patients (3). One of the solutions for clinical readiness of nurses is scientific care based on standard care models (8). On the other hand, using nursing theories in studies is regarded as an attempt for validating nursing theories, organizing nursing measures, and generating knowledge (9). Thus, diagnosis and training self-care methods and adaptation with current status, which eliminate or reduce fatigue in these patients, would be highly effective (10). Overall, various models and theories have been proposed in relation to adaptation with chronic diseases (11). Roy model is one of the nursing models that were designed for better understanding of adaptation concept (12). Based on Roy model, the human being is a biological, psychological, social, and spiritual creature, who is connected to his surrounding environment and follows adaptation.

mechanisms for preserving balance (13). “Roy” introduces this model as follows: in order to achieve this goal, the patient requires to achieve physical adaptation (physiologic dimension) and psychological adaptation in different dimensions (self-concept, role function, interdependence) (14). According to this model, the nurse systematically and carefully investigates the patient through interview, observation, and measurement. Then, she specifies the maladaptive behavior, which is in fact the patient's problem, in four dimensions, along with the stimulus of behavior (causes), and subsequently, designs the precise educational and care plans to address the patient's problems (maladaptive behaviors) (11). In this regard, the studies have also indicated a positive effect of using Roy adaptation model on controlling the disease in chronic diseases (14). For example, the study of Bakan and Akyol reported that using Roy model caused increased adaptation in patients with heart failure (15). Researchers used Roy's Adaptation Theory in cardiac patients as a suitable tool for patient care and performing nursing measures (16).

Considering the above-mentioned material, fatigue and reduced ability or inability of patients with heart failure in performing daily activities are raised as maladaptive behaviors, which require nursing attention. Thus, diagnosis and training effective adaptation methods in this regard would be valuable. On the other hand, searches in various databases imply lack of application of this model on fatigue and daily activities of patients with heart failure in Iran. The current study can be regarded as a small step in application of this model in practice. Therefore, in order to achieve this goal, the current study was designed so that the effect of educational plan based on Roy adaptation model on fatigue and daily activities of patients with heart failure is measured.

## Methods

### Sample and recruitment:

To determine the sample size for the first time as a pilot, ten patients in the control group and ten patients in the intervention group were evaluated using the obtained results, and the formula for comparing the mean of the final sample rates with confidence of 95%, 80% of the 30 people in each group was calculated. Samples were selected through convenience sampling method and then randomly assigned into intervention (n = 30) and control groups (n = 30). The inclusion criteria included all patients diagnosed with heart failure and hospitalized and treated for their cardiac disease; they had full consciousness and their conditions were not acute; subjects had not previously received any kind of formal training related to this research; they had not been suffering from chronic and other debilitating diseases and none of the patients was of the healthcare team members. The exclusion criteria included absenteesm for more than two sessions, and unwillingness to continue to participate in the study.

### Instrument and data collection:

The first questioner included questions about demographic characteristics of participants including age, gender, marital status, and educational level, number of hospitalization, job and duration of disease.

### Roy Adaptation Model (RAM):

To formulate a care and educational plan in the intervention group, Roy model understanding form was used. The form was designed by Roy to examine maladaptive behaviors in four modes of physiologic (50 questions), self-concept (22 questions), role function (10 questions) and (8 questions) for interdependence of the patient mode (17).

The content validity of the questionnaire was approved by 10 professors of the Kerman University of Medical Sciences. The form for the verification of Roy model, which is a standard form, was confirmed in the study (14) by using test-retest reliability method with correlation coefficient of 0.75. In this study, Cronbach's alpha method was used for determining reliability, and reliability coefficient was reported as 0.85.

### Piper Fatigue Scale (PFS):

The four-dimensional fatigue scale (Behavioral/severity, Emotional, Sensory and Cognitive) consists of 27 items. Items 2–23 are calculated as eleven points out of which the total average score is based between 0-10. The higher score denotes higher level of fatigue. In addition, five end items are included for enrichment of the questionnaire, which are not calculated. The content validity of the questionnaire was approved by 10 professors of the Kerman University of Medical Sciences. Reliability of this tool was confirmed in the study by (18) with Pearson correlation coefficient as 0.08. In the current research, correlation was confirmed as 0.80 using Cronbach's alpha.

### Barthel Index of Activities of Daily Living (BIADL):

This scale was used to measure the performance of the patients. It had 28 items that measure one's ability in nutrition, feeding, mobility, intestinal and bladder control, using wheelchair, bed to chair transfer and vice versa, bathing, and wearing cloth. Maximum score is 112 and minimum score is 0. Scores below 20 indicate total dependency and 100 denotes independence. Higher scores denote optimal status. Reliability of the tool was confirmed in study by (17). The content validity of the questionnaire was approved by 10 professors of the Kerman University of Medical Sciences. Reliability of this tool in the current research was confirmed as 0.82 using Cronbach's alpha.

### Intervention and data collection:

The intervention group received the care plan base Roy adaptation model of through a face-to-face training program in 4 sessions and group training program in 2 sessions at one month. Training program in intervention group base of Roy adaptation model in physiologic dimension for example included education appropriate and healthy nutrition, non-elimination of breakfast, adequate water drinking during the day, recommendation to eat fruits and vegetables, training required mobility, and factors affecting sleeping. In self-concept, education would be in line with creating positive changes in mental images and ideal self, in interrelationships dimension, education would be participation in religious discussions and ceremonies, and in role-playing dimension, education would be training how to participate in social ceremonies, and peer groups.

In addition, the main points of the sessions were provided with the patients in written form. The researcher followed up implementation of educational plan by the patients (intervention duration) through presence in the hospital and phone calls, and answered questions from patients. Interval followed by a follow-up period for 4 weeks later, while the control group only received the regular services from hospital.

### Ethics consideration:

This study was approved by the Ethical Committee of the Islamic Azad University, Yazd Branch, prior to the collection of data. The ethical approval code is IR.IAU.YAZD.REC.1396.8. Research data were collected once research objectives had been explained to qualified participants and informed consents was signed. The confidentiality of information and the possibility of leaving were also ensured. Patients were told that they had the right to withdraw at any time throughout the study.

### Data analyses:

Data were analyzed using SPSS version 20 software (IBM Corp, Armonk, New York). The Kolmogorov-Smirnov Test was used to study normal distribution of data. Because the data distribution was normal, parametric tests were used, including Pearson correlation coefficient, independent t-test, paired t-test, Chi-square and ANOVA. A significance level of *P* ≤ .05 was considered for all tests.

## Results

The finding showed that the majority of the participants (71.9%) in both groups were within the age range of 45–60. In the both groups, 62.7% were females and the majority of the participants' (58.7%) base of education level in both groups was bachelor degree; other demographic characteristics are shown in Table 1. The Chi-square test showed that the intervention and control groups were not significantly different according to demographic variables, P < 0.05, (Table 1).

**Table 1:** Distribution of demographic variables of research units in intervention and control groups.

Variable	Control group	Intervention group N (%)	P
	N (%)		
Age			
25–35	6(20.0)	5(16.6)	
35–45	8(26.6)	10(33.3)	0.85
45–60	16(53.3)	15(50.0)	
Gender			
Male	12(40.0)	14(46.6)	0.64
Female	18(60.0)	16(53.3)	
Marital status			
Single	11(36.6)	11(36.6)	0.87
Married	19(63.3)	19(63.3)	
Frequency of Hospitalization in Month			
Once	12(40.0)	8(26.6)	
Twice	18(60.0)	15(50.0)	0.06
Three and more	0(0)	7(23.3)	
Job			
Worker	6(20.0)	2(6.6)	
Employee	14(46.6)	16(53.3)	0.06
Housewife	10(33.3)	12(40.0)	
Education level			
Diploma	5(16.6)	10(33.3)	
Bachelor	20(66.6)	16(53.3)	0.51
Master	5(16.6)	4(13.33)	
Duration of disease (Years)			
1–5	20(16.0)	15(50.0)	
5–10	7(20.0)	10 (20.0)	0.32
10–20	3(20.0)	5(33.3)	

The findings indicated that educational plan based on Roy adaptation model can influence maladaptive behaviors of patients with heart failure in all physiologic, self-concept, role function and interdependence modes, so that significant reduction was observed in the number of maladaptive behaviors after intervention compared to before intervention.

Results of the current research showed that in relation with mean score, fatigue and daily activities of life were not significant in intervention and control groups before intervention, while it was significant after intervention, P < 0.05. Paired t-test showed that mean score of fatigue and daily activities in intervention group before and after intervention was significant (P < 0.01), so that in the intervention group, the mean score of fatigue was significantly decreased after intervention (3.5 ± 1.7) compared to before intervention (6.2 ± 2.2). Also, in the intervention group, the mean score of daily activities was significantly increased after intervention (62.2 ± 12.8) compared to before intervention (45.3 ± 11.2), as shown in Table 2. T-test showed a significant difference between intervention and control groups in terms of mean score of fatigue and daily activities, so that the intervention group had significantly lower mean scores in fatigue and higher daily activities after intervention compared to the control group, P < 0.01, shown in Table 3.

**Table 2:** Comparing fatigue and daily activities before and after intervention in case and control group.

Variable	Intervention	Control group		Intervention group		p
		Mean	SD	Mean	SD	
**Fatigue**	Before	6.5	1.9	6.3	2.2	0.54
	After	7	1.95	3.5	1.7	0.001
**Daily activities**	Before	49.5	12.5	45.3	11.2	0.22
	After	47.6	10.5	62.2	12.8	0.001

**Table 3:** Comparing difference in mean score of fatigue and daily activities before and after intervention in two groups.

Variable	Group	Before and after intervention	p
**Fatigue**	Control group	0.3±1.02	< 0.01
	Intervention group	2.8±1.8	
**Daily activities**	Control group	1±6.5	< 0.01
	Intervention group	14.8±8.9	

The ANOVA test showed that there was a significant relationship between the duration of time of heart failure disease and frequency of hospitalization with fatigue in both intervention and control groups, *P* ≤ .05, so that the mean score of fatigue was less than other participants in patients hospitalized twice a month because of the nature of the disease and their duration of heart failure was 5 years or less. The ANOVA test showed that there was a significant relationship between the duration of time of heart failure disease and frequency of hospitalization with daily activities level, *P* ≤ .05, so that the mean score of daily activities level in both the control and the intervention groups was more than other participants in the first 5 years of heart failure and hospitalization of twice per month. The result showed that no significant relationship was observed between other demographic variables including age, gender, marital status, job and education with fatigue and daily activities variables in both groups.

## Discussion

It is clear that adaptation of patients with the problems and long-term complications of disease play effective and significant roles in controlling the disease and promoting qualitative level of their life in all chronic diseases (14,19). Research findings indicated that educational plan based on Roy adaptation model can influence maladaptive behaviors of patients with heart failure in all physiologic, self-concept, role function and interdependence modes, so that significant reduction was observed in the number of maladaptive behaviors after intervention compared to before intervention.

This finding is in line with studies conducted by studies of (20–22).They revealed that the mean score of all adaptation dimension of Roy adaptation model significantly increased after intervention. However, this finding is not in line with studies conducted by (14,18) that the cause can be difference in research tools and cultural components. Patients with heart failure are required to perform some activities, pursue care recommendations, and change life style in order to control and avoid consequences of the disease. Thus, involving patients in implementation of these adaptation behaviors as well as change and manipulation of stimulus of maladaptive behaviors is an important factor in adaptation with heart failure disease. Therefore, it can be said Roy model of adaptation is one of the most widespread nursing models in coming to terms with diverse illnesses and problems.

The findings of this research showed effectiveness of the proposed educational plan in improvement of patients' ability in performing daily activities in intervention group, so that significant statistical difference was observed in intervention group before and after intervention while no significant statistical difference was observed in the control group before and after intervention. This finding is in line with the study conducted by (19) with titled using Roy model on daily activities of hemodialysis patients, the study of (23) conducted on daily activities of patients with MS using self-care program based on Orem's model and the study of (24) with title effects of antenatal education on maternal prenatal and postpartum adaptation. They found that using nursing theories has a positive effect on increasing ability to perform daily activities of patients' lives. The patients' ability to perform daily life activities is one of the important indicators in assessing the role of performance compatibility, the results of this study also indicate the effectiveness of the provided educational plan, in improving the patient's ability to perform life daily activities in the intervention group.

In the present study, the results showed that the mean Piper fatigue score in the pre and post intervention stage was significantly different in the intervention group in behavioral, emotional, sensory, and cognitive aspects of Piper's fatigue scale, so that the mean score of fatigue in each aspect was decreased in intervention group, while no difference was observed in control group. This finding is in line with studies conducted by (4,10,23,25,26). Various methods have been investigated for reducing fatigue in patients with heart failure. Including music and back massage (7), energy conservation techniques (3) and acupressure (1), although these methods have helped patients with heart failure, the nature of these methods should be taken into consideration.

That is, the more they are derived from their needs, motivation and empathy are increased in patients and families and empowers them. The present study also indicated that the implementation of a educational program based on Roy adaptation model, adapted from their educational and tangible needs, is significantly effective on the fatigue of patients.

The results of this study showed that there was a significant relationship between the duration of time of heart failure disease and frequency of hospitalization with fatigue in both intervention and control groups so that in patients hospitalized twice a month, with heart failure duration of 5 years or less, the mean score of fatigue was less than other participants.

This result was consistent with similar studies done in other chronic patients such as the studies of (18,27). They also reported in their studies that the more the frequency of hospitalization, the more the fatigue of individuals. According to the high prevalence of fatigue in heart failure patients because of the nature of disease, it is important to provide necessary measures and facilities by health authorities in order to provide community-based therapeutic care, especially at home, to prevent multiple hospitalizations and increase well-being in these people.

The results of this study showed that there was a significant relationship between the duration of time of heart failure disease and frequency of hospitalization with daily activities level, so that the mean score of daily activities level in both control and intervention groups was more than other participants in the first 5 years of heart failure and hospitalization twice per month. This result was consistent with the results of studies by (28,29). Therefore, the nurses and patients' family must perform necessary interventions in order to increase daily life activities level and appropriate lifestyle patterns by increasing the disease duration through education. There was no significant relationship between other demographic features of the participants with daily activities level and fatigue in both control and intervention groups. In general, the absence of a significant relationship between some demographic information and fatigue does not mean that there is no real relationship, but the sample size of this study was not estimated to investigate these relationships. More specific studies with appropriate sample size and focus on these variables are needed to investigate these relationships.

The limitations of this study include its small sample size, reluctance of some patients to participate in the research, and lack of regular attendance on the part of some participants due to occupational reasons. Since these restrictions may affect generalizability of the findings and constrain their applicability, it is suggested that future studies consider larger sample sizes. We also suggest further studies with more long-term follow-ups.

Roy's nursing model allows identification of maladaptive behaviors in different aspects and comprehensive educational planning based on the needs and stimuli of maladaptive behaviors of patient because of having need-assessment approach. In addition, this model which is a noninvasive, non-pharmacological, low-cost and comprehensive method, can be used for clinical care and training chronic patients including patients with heart failure.

Given the findings and discussions, research findings suggest that education based on Roy adaptation model can influence reduction of fatigue and promotion of daily activities of patients with heart failure. Thus, it is suggested that using Roy model is considered for clinical care.
